# Relative Contributions of Norepinephrine and Serotonin Transporters to Antinociceptive Synergy between Monoamine Reuptake Inhibitors and Morphine in the Rat Formalin Model

**DOI:** 10.1371/journal.pone.0074891

**Published:** 2013-09-30

**Authors:** Fei Shen, Pamela R. Tsuruda, Jacqueline A. M. Smith, Glenmar P. Obedencio, William J. Martin

**Affiliations:** 1 Departments of Pharmacology, Theravance Inc., South San Francisco, California, United States of America; 2 Departments of Molecular and Cell Biology, Theravance Inc., South San Francisco, California, United States of America; 3 Departments of Drug Metabolism and Pharmacokinetics, Theravance Inc., South San Francisco, California, United States of America; University of Kentucky Medical Center, United States of America

## Abstract

Multimodal analgesia is designed to optimize pain relief by coadministering drugs with distinct mechanisms of action or by combining multiple pharmacologies within a single molecule. In clinical settings, combinations of monoamine reuptake inhibitors and opioid receptor agonists have been explored and one currently available analgesic, tapentadol, functions as both a µ-opioid receptor agonist and a norepinephrine transporter inhibitor. However, it is unclear whether the combination of selective norepinephrine reuptake inhibition and µ-receptor agonism achieves an optimal antinociceptive synergy. In this study, we assessed the pharmacodynamic interactions between morphine and monoamine reuptake inhibitors that possess different affinities and selectivities for norepinephrine and serotonin transporters. Using the rat formalin model, in conjunction with measurements of *ex vivo* transporter occupancy, we show that neither the norepinephrine-selective inhibitor, esreboxetine, nor the serotonin-selective reuptake inhibitor, fluoxetine, produce antinociceptive synergy with morphine. Atomoxetine, a monoamine reuptake inhibitor that achieves higher levels of norepinephrine than serotonin transporter occupancy, exhibited robust antinociceptive synergy with morphine. Similarly, a fixed-dose combination of esreboxetine and fluoxetine which achieves comparable levels of transporter occupancy potentiated the antinociceptive response to morphine. By contrast, duloxetine, a monoamine reuptake inhibitor that achieves higher serotonin than norepinephrine transporter occupancy, failed to potentiate the antinociceptive response to morphine. However, when duloxetine was coadministered with the 5-HT_3_ receptor antagonist, ondansetron, potentiation of the antinociceptive response to morphine was revealed. These results support the notion that inhibition of both serotonin and norepinephrine transporters is required for monoamine reuptake inhibitor and opioid-mediated antinociceptive synergy; yet, excess serotonin, acting via 5-HT_3_ receptors, may reduce the potential for synergistic interactions. Thus, in the rat formalin model, the balance between norepinephrine and serotonin transporter inhibition influences the degree of antinociceptive synergy observed between monoamine reuptake inhibitors and morphine.

## Introduction

The effectiveness of clinical pain management can often be improved by co-administering agents that leverage different pharmacological mechanisms or by combining multiple pharmacologies within a single molecule. The basis for this multimodal analgesia is informed by improved understanding of the endogenous substrates of pain and analgesia. Serotonin (5-HT) and norepinephrine (NE), along with opioids, are the principle endogenous substrates in the descending pain modulatory pathway, and concurrent modulation of their activity provides a rational approach to analgesic combination therapy [1]–[6]. The potential for improved pain management through concurrent targeting of these different mechanisms is exemplified by tapentadol, a dual µ-opioid receptor agonist and norepinephrine transporter (NET) inhibitor [7]–[10]. Tapentadol demonstrates similar analgesic efficacy to oxycodone, but the improved gastrointestinal side effect profile is consistent with an opioid-sparing effect [11]. An alternate approach to multimodal analgesia is to co-administer compounds that confer analgesic efficacy via the different mechanisms of action, such as gabapentinoids, nonsteroidal anti-inflammatory drugs (NSAIDs), tricyclic antidepressants (TCAs), monoamine reuptake inhibitors and opioids [12]–[15]. While the use of combination therapy of monoamine reuptake inhibitors and morphine to achieve multimodal analgesia is common in clinical practice [9], [13], [14], [16], the precise pharmacological profile of monoamine reuptake inhibitors that will provide the optimal degree of analgesic synergy when combined with morphine remains to be determined. Strong preclinical and clinical evidence exists for synergistic effects between inhibition of NET and opioid receptor activation [13], [14], [16]–[21]. The potential for serotonin transporter (SERT) inhibition to modulate opioid-induced analgesia is, however, more controversial [14], [21]–[23].

The objective of the present study was to determine the influence of the balance of NET and SERT inhibition on the apparent antinociceptive synergy between monoamine reuptake inhibitors and morphine. Using the rat formalin model in conjunction with measurements of *ex vivo* transporter occupancy, our study was designed to demonstrate, quantitatively, whether the balance between NET and SERT inhibition influences the synergistic interaction between parenteral administration of monoamine reuptake inhibitors and morphine. The rat formalin model of injury-evoked inflammatory pain was selected for these studies as there is evidence that the monoaminergic descending inhibitory systems are significantly activated [24], and that this endogenous inhibitory system can be augmented by treatment with a monoamine reuptake inhibitor (e.g., duloxetine) [25]. In addition, the reproducibility, sensitivity to different classes of clinically-validated analgesics, and high throughput of the formalin model make it ideally suited to probe potential synergistic interactions with combination therapy [26], [27]. Our findings suggest that the inhibition of both SERT and NET is required for morphine-mediated antinociceptive synergy, but excessive serotonin transporter inhibition may counteract with this interaction by activating 5-HT_3_ receptors. Thus, the balance of reuptake inhibitor activity at NE and 5-HT transporters can influence manifestation of antinociceptive synergy with opioids in the rat formalin model.

## Materials and Methods

### 2.1. Animals

Adult male Sprague-Dawley rats (Harlan, Livermore, CA, 150–220 g) were housed in pairs in an AALAAC accredited animal care facility on a 12-h light/dark cycle and were given free access to food and water. All experiments were approved by the Theravance Institutional Animal Care and Use Committee and adhered to guidelines established by the International Association for the Study of Pain.

### 2.2. Materials

Esreboxetine, duloxetine and fluoxetine were purchased from Waterstone Technology LLC (Carmel, IN), ondansetron from Tocris (Ellisville, MO), atomoxetine from AK Scientific (Mountain View, CA), and formalin, morphine and naloxone from Sigma-Aldrich (St. Louis., MO).

### 2.3. *In vitro* Radioligand Binding

Determination of *in vitro* apparent binding affinity (pK_i_ values) was performed as described previously [28]. Frozen rat cortical tissue was homogenized in buffer containing Tris (10 mM) and EDTA (1 mM) and centrifuged at 3,400×g. The membrane pellet was obtained by centrifugation of the supernatant at 40,000×g and was resuspended in Tris (50 mM) buffer with sucrose (10%). Membranes (12.5–25 µg protein) were incubated at room temperature for 6 h in the presence of [^3^H]-citalopram (PerkinElmer, Waltham, MA) or [^3^H]-nisoxetine (GE Healthcare Life Sciences, Piscataway, NJ or PerkinElmer) and the compound of interest (10 pM–100 µM) prior to assay termination by rapid filtration.

### 2.4. *In vitro* Neurotransmitter Uptake

Determination of SERT and NET inhibitory potencies (pIC_50_ values) was performed as described previously [28]. Freshly dissected cortical tissue was homogenized in sucrose buffer containing: sucrose (320 mM), HEPES (10 mM), ascorbic acid (200 µM), and pargyline (200 µM) and centrifuged at 1,000×g. The crude synaptosomal pellet was obtained by centrifugation of the supernatant at 10,000×g and was resuspended in sucrose buffer. Synaptosomes (10 µg protein) were pre-incubated at 37°C for 30 min with test compound (10 pM–100 µM) then incubated for 6 min at 37°C with [^3^H]-NE (40 nM; GE Healthcare Life Sciences) or [^3^H]-5-HT (20 nM; Perkin Elmer) prior to assay termination by rapid filtration.

### 2.5. Rat Formalin Model of Nociception

Compounds were assessed for their ability to inhibit the behavioral response evoked by a 50 µL injection of formalin (5%) as described previously [29]. The monoamine reuptake inhibitors, ondansetron (5-HT_3_ antagonist), naloxone (µ-opioid antagonist), or vehicle (10% Tween-20 in distilled water) were administered intraperitoneally (IP), while morphine was dosed subcutaneously (SC). Formalin was injected into the dorsal surface of the right hind paw 30 min after administration of test compounds, and the number of flinches was counted continuously using an automated nociception analyzer (UCSD Anesthesiology Research, San Diego, CA). The antinociceptive period was determined 15–40 min post-formalin injection (termed phase 2A). The total number of flinches during phase 2A was used to quantify the antinociceptive response, as it directly reflects the supraspinal inhibitory mechanisms that dampen or reduce the excitation of dorsal horn neurons immediately following an acute noxious stimuli [24], [30]. Following completion of behavioral testing in the formalin model (i.e., 75 min post-dosing with either test compound or vehicle), rats were euthanized for *ex vivo* transporter occupancy or pharmacokinetic studies.

### 2.6. *Ex vivo* Transporter Occupancy

*Ex vivo* occupancy studies were performed similarly to those described previously [31]. At a single time point at the conclusion of phase 2A (75 min post-dose), a 5 mm^2^ piece of frontal cortex was dissected, frozen rapidly, and stored at −80°C until use. Cortical crude homogenates were prepared and the initial rates (v_i(vehicle)_ or v_i_, respectively) of [^3^H]-citalopram (SERT) or [^3^H]-nisoxetine (NET) binding determined over a 3-min time course. The % NET or SERT transporter occupancy for compound-dosed animals was calculated using the following equation: 100*(1−v_i_)/average v_i(vehicle)._

### 2.7. RotaRod Test

Motor coordination was assessed using an accelerating RotaRod. One day prior to test compound administration, rats were trained to walk on a 6 cm rotating rod of constant speed (10 rpm) (UGO Basile, 7750). For the testing phase, animals were placed on the rod (initial rotational speed of 10 rpm), 60 min after atomoxetine (IP) or/and morphine (SC) administration. The RotaRod then accelerated, at a rate of 10 rpm up to maximal 40 rpm, and total walking time (latency to fall from the rod) was recorded.

### 2.8. Pharmacokinetic and Bioanalytical Analysis

Plasma and brain concentrations of reuptake inhibitors were determined by LC/MS/MS. Samples (10–20 µL) were injected in a Hypurity C18 column (50×2.1 mm; 3 µM) with a flow rate of 0.5 mL/min. Mobile phase A consisted of 0.2% formic acid in water or 0.2% formic acid in 95% water and 5% acetonitrile while mobile phase B consisted of 0.2% formic acid in acetonitrile or 0.2% formic acid in 95% acetonitrile and 5% water. Various gradient elutions were used and the mass spectrometers (Sciex API5000 and API4000; Applied Biosystems, Foster City, CA) were operated in positive ion multiple reaction monitoring mode. The lower limit of quantification for all four compounds was between 0.125 and 5 ng/mL in plasma or 0.5 and 20 ng/g in brain.

### 2.9. Protein Binding in Rat Plasma and Brain Homogenates

The *in vitro* unbound fraction of reuptake inhibitors in rat brain homogenates (5 µM) and in rat plasma protein (1 µM) was evaluated using equilibrium dialysis using the HT-Dialysis device (Gayles-Ferry, CT). Brain homogenate or plasma was spiked with test compounds and dialyzed against blank PBS for 5 hours at 37°C. The matrices from both sides of the dialysis membranes were equalized and extracted with acetonitrile. Unbound fractions for each compound were calculated as the ratio of peak areas from the PBS side to peak areas from the tissue or plasma side. Peak areas were corrected for dilution of the tissue made prior to spiking compound. Quantification was via LC/MS/MS.

### 2.10. Data Analysis and Statistics

Radioligand binding and neurotransmitter uptake data were analyzed by nonlinear regression analysis as described previously [28]. Data are expressed as pK_i_ or pIC_50_ (negative decadic logarithm K_i_ or IC_50_, respectively) values (mean ± standard deviation). The selectivities for uptake inhibition (rounded to one significant figure) for NET or SERT were determined as shown in the following example for NET: selectivity = 10^(pIC50 at NET-pIC50 at SERT).^

For the rat formalin model, the percent inhibition of flinching was determined by comparing the total number of flinches during phase 2A to concurrently tested vehicle-treated rats according to the following formula: (Vehicle – Treatment)/(Vehicle) ×100%. The efficacious doses of each compound were defined as those yielding statistically significant inhibition of flinching compared to vehicle treatment (One-way analysis of variance (ANOVA) with *post hoc* Dunnett’s multiple comparisons, p<0.05, data not shown). Subefficacious doses of morphine (1 mg/kg, SC), atomoxetine (3 and 10 mg/kg, IP), duloxetine (5 mg/kg, IP), esreboxetine (10 mg/kg, IP) and fluoxetine (10 mg/kg, IP) were defined accordingly. The percent inhibition of flinching for each rat at each dose was used to generate ED_50_ values determined by a sigmoidal dose-response (variable slope) curve with minima and maxima constrained to 0 and 100, respectively (GraphPad™ Prism). Data were represented as mean ± SEM for each treatment group. To evaluate potential additive or synergistic interactions, fixed-dose combination and fixed-ratio combination experimental designs were used as described below.

#### Fixed-dose design

ED_50_ values were determined by generating dose-response curves for morphine or monoamine reuptake inhibitors alone, and in the presence of a subefficacious dose of monoamine reuptake inhibitors or morphine, respectively. Potential additive or synergistic interactions between the pairs of test compounds were evaluated by comparing the dose-response curves for morphine in the absence and presence of a fixed-dose of monoamine reuptake inhibitor (or *vice versa*). A shift in the ED_50_ values, with non-overlapping 95% confidence intervals (CI), was deemed a statistically significant effect.

#### Fixed-ratio design

ED_50_ values were determined by generating dose-response curves for atomoxetine alone and for fixed-ratio combinations with morphine. Isobolographic analysis was used to evaluate mathematically whether co-administration of the two drugs produced an additive, less than additive (i.e., antagonistic) or more than additive (i.e., synergistic) antinociceptive effect in rat formalin model [32]. The ED_50_ of each drug (Drug A and Drug B) alone was plotted on x- and y-axes respectively, and a connecting line between the two ED_50_s was drawn (line of additivity). The theoretical additive ED_50_ of the pair was calculated using the equation, F×ED_50 Drug A_+(1–F) ×ED_50 Drug B_. F is a fractional multiplier related to the ratio of drug A and B in the mixture (mixture ratio = concentration of drug A/concentration of drug B) where F = mixture ratio/(mixture ratio+ED_50 Drug A/_ED_50 Drug B_). If an observed ED_50_ (with 95% CI) of a fixed-ratio combination of the pair lay to the left of, and below, the theoretical additive ED_50_ (with 95% CI), then the interaction of the two drugs was deemed synergistic [33].

For the pharmacokinetic and bioanalytical analyses, free plasma and free brain concentrations were calculated using the *in vitro* unbound fractions in rat plasma and brain homogenates, respectively. The unbound fraction in rat plasma for duloxetine, atomoxetine, fluoxetine and esreboxetine are 0.031, 0.18, 0.067 and 0.25, respectively. The unbound fraction in rat brain homogenate for duloxetine, atomoxetine, fluoxetine and esreboxetine are 0.007, 0.021, 0.0045 and 0.053, respectively (Theravance, Inc., unpublished data).

## Results

### 3.1. *In vitro* Pharmacological Profile of Monoamine Reuptake Inhibitors

The *in vitro* pharmacological profiles of monoamine reuptake inhibitors were determined in radioligand binding and neurotransmitter uptake assays using rat native SERT and NET. All compounds exhibited concentration-dependent inhibition of [^3^H]-citalopram and [^3^H]-nisoxetine binding in rat cortical membranes, as well as [^3^H]-5-HT and [^3^H]-NE uptake into synaptosomal preparations. Fluoxetine and duloxetine demonstrated selectivity for SERT over NET (50-fold and 5-fold SERT-selective, respectively, based on uptake inhibition, pIC_50_ values), while atomoxetine and esreboxetine were NET-selective (30-fold and 20,000-fold, respectively) (Table 1). A similar rank order of selectivities was calculated using apparent binding affinities (pK_i_) (Table 1). This rank order was consistent with previously reported selectivities for the human transporters [28].

**Table 1:** *In vitro* uptake inhibitory potency (pIC_50_) and apparent binding affinity (pK_i_) of fluoxetine, duloxetine, atomoxetine and esreboxetine in rat cortical membrane or synaptosomal preparations, respectively (n = 3–12).

Compound	pIC_50_			pK_i_	
	RatSERT	RatNET	Transporterselectivity	RatSERT	RatNET
Fluoxetine	7.8±0.1	6.1±0.1	50-fold SERT	8.8±0.1	6.3±0.3
Duloxetine	9.1±0.1	8.4±0.2	5-fold SERT	10.0±0.1	8.4±0.1
Atomoxetine	7.1±0.1	8.6±0.2	30-fold NET	7.8±0.1	8.7±0.2
Esreboxetine	5.3±0.1	9.6±0.2	20,000-fold NET	5.6± <0.1	9.2±0.1

### 3.2. Neither Esreboxetine Nor Fluoxetine Alone Enhances Morphine-induced Antinociception

**Figure 1:**
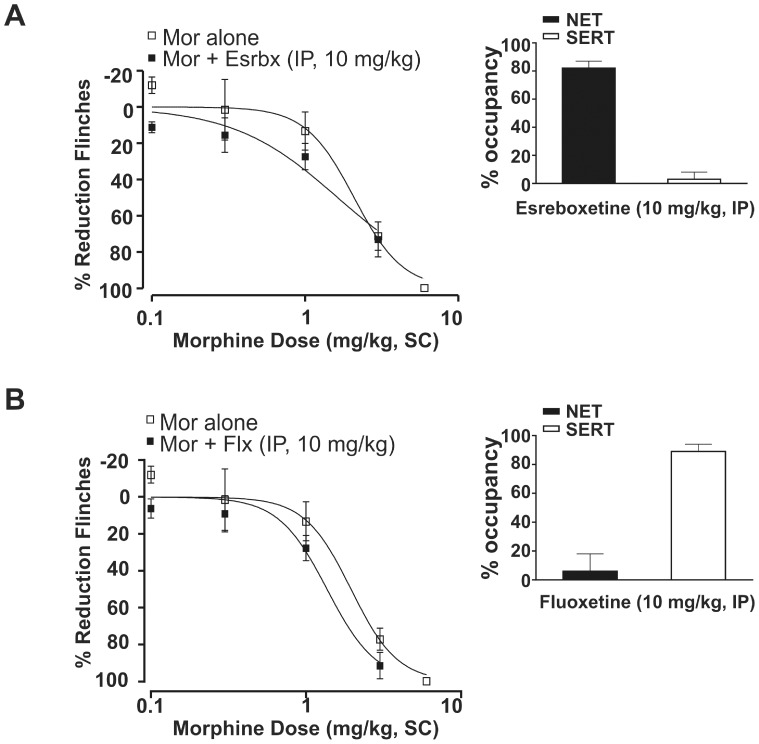
Neither esreboxetine nor fluoxetine exhibited antinociceptive synergy with morphine in the rat formalin model. (A) The selective NET inhibitor esreboxetine alone (Esrbx, IP, 10 mg/kg), failed to shift the morphine (Mor) dose-response curve (n = 6). Morphine alone: ED_50_ = 2.0 mg/kg (95% CI: 1.8–2.5); morphine+ esreboxetine (IP, 10 mg/kg): ED_50_ = 1.6 mg/kg (95% CI: 1.1–2.4). All data points are shown as mean ± SEM for each group and are expressed as percentage of controls. Inset (A) Esreboxetine (IP, 10 mg/kg) was associated with 82±5% NET and 3±5% SERT occupancy measured *ex vivo* at 75 min post-dose. All occupancy data represent mean (± SEM) for each group. (B) The selective SERT inhibitor fluoxetine alone (Flx, IP, 10 mg/kg), failed to shift the morphine dose-response curve (n = 6). Morphine+fluoxetine (IP, 10 mg/kg): ED_50_ = 1.6 mg/kg (95% CI: 1.2–2.2). Inset (B) Fluoxetine (IP, 10 mg/kg) was associated with 6±12% NET and 89±5% SERT occupancy measured *ex vivo* at 75 min post-dose.

Morphine produced dose-dependent antinociception in the rat formalin model; the ED_50_ of morphine was 2.0 mg/kg (95% CI: 1.8–2.5; Fig. 1).

To investigate whether NET or SERT inhibition alone is sufficient for antinociceptive synergy, the selective NET inhibitor esreboxetine or selective SERT inhibitor fluoxetine was each tested in combination with morphine. Neither 10 mg/kg (IP) esreboxetine nor 10 mg/kg (IP) fluoxetine alone produced antinociception in the rat formalin model (17±9% and 9±4%, respectively). At these doses, the corresponding transporter occupancies measured *ex vivo* at the conclusion of phase 2A were: 82±5% NET and 3±5% SERT for esreboxetine, 6±12% NET and 89±5% SERT for fluoxetine, respectively (Figs. 1A inset and 1B inset). Coadministration of esreboxetine and morphine, or fluoxetine and morphine, did not produce a significant leftward shift in the morphine dose-response curve (Figs. 1A & B; ED_50_ = 1.6 mg/kg with 95% CI: 1.1–2.4 and ED_50_ = 1.6 mg/kg with 95% CI: 1.2–2.2, respectively).

### 3.3. Dual NET and SERT Inhibition Enhances Morphine-induced Antinociception

**Figure 2:**
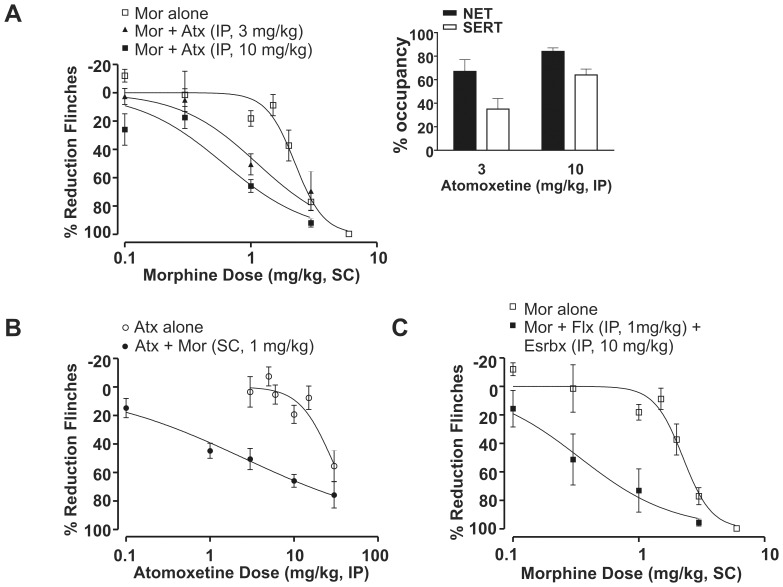
Atomoxetine exhibited antinociceptive synergy with morphine using a fixed-dose design in the rat formalin model. (A) Both 3 and 10 mg/kg atomoxetine (Atx, IP) shifted the morphine (Mor) dose-response curve leftward in the rat formalin model (n = 6–16). Morphine alone: ED_50_ = 2.3 mg/kg (95% CI: 2.0–2.5); morphine+atomoxetine (IP, 3 mg/kg): ED_50_ = 1.1 mg/kg (95% CI: 0.8–1.6); and morphine+atomoxetine (IP, 10 mg/kg): ED_50_ = 0.6 mg/kg (95% CI: 0.4–0.8). All data points are shown as mean ± SEM for each group and are expressed as percentage of controls. Inset (A) Atomoxetine (IP) at 3 and 10 mg/kg was associated with 67±10% and 84±3% for NET and 35±9% and 64±5% for SERT occupancy measured *ex vivo* at 75 min post-dose, respectively. All occupancy data represent mean (± SEM) for each group. (B) A subefficacious dose of morphine 1 mg/kg (SC) left-shifted the atomoxetine dose-response curve (n = 6–16). Atomoxetine alone: ED_50_ = 27.8 mg/kg (95% CI: 22–36); and atomoxetine+morphine (SC, 1 mg/kg): ED_50_ = 2.5 mg/kg (95% CI: 1.3–4.7). (C) A fixed combination of NET selective inhibitor esreboxetine (Esrbx, IP, 10 mg/kg) and SERT selective inhibitor fluoxetine (Flx, IP, 1 mg/kg) left-shifted the morphine dose-response curve (n = 6–12). Morphine alone: ED_50_ = 2.3 mg/kg (95% CI: 2.0–2.5); morphine+esreboxetine (IP, 10 mg/kg)+fluoxetine (IP, 1 mg/kg): ED_50_ = 0.3 mg/kg (95% CI: 0.2–0.7).

To explore whether concurrent inhibition of norepinephrine and serotonin transporters enhances antinociception with morphine, increasing doses of atomoxetine were tested alone and in combination with morphine. Increasing doses of morphine were also tested alone and in combination with atomoxetine. The ED_50_ of morphine dose-response curve alone was 2.3 mg/kg (95% CI: 2.0–2.5; Fig. 2A); whereas, the ED_50_ of atomoxetine was 27.8 mg/kg (95% CI: 22–36; Fig. 2B).

At doses of 3 and 10 mg/kg, atomoxetine did not significantly reduce flinching behavior (reductions of 4±11% and 19±6%, respectively; Fig. 2B). At these subefficacious doses, the corresponding transporter occupancies were: 67±10% and 84±3% for NET and 35±9% and 64±5% for SERT, respectively (Fig. 2A inset). Using a fixed-dose experimental design, atomoxetine at 3 and 10 mg/kg produced a leftward shift in the morphine dose-response curve, consistent with -potentiation of the antinociceptive effect of morphine (Fig. 2A; ED_50_ = 1.1 mg/kg with 95% CI: 0.8–1.6 and ED_50_ = 0.6 mg/kg with 95% CI: 0.4–0.8, respectively). Conversely, in the presence of a subefficacious dose of morphine (1 mg/kg, SC), which alone yielded 18±5% inhibition of flinching behavior, there was an approximate 10-fold leftward shift in - the atomoxetine dose-response curve (Fig. 2B; ED_50_ = 2.5 mg/kg with 95% CI: 1.3–4.7). The apparent potentiation of atomoxetine-induced antinociception by morphine does not reflect a pharmacokinetic interaction as the plasma _unbound_ and brain_ unbound_ concentrations of atomoxetine were not significantly different between rats administered the monoamine reuptake inhibitor alone and those administered the inhibitor in combination with 3 mg/kg SC morphine (Table 2).

**Table 2:** Plasma _unbound_ and brain_ unbound_ concentrations of atomoxetine in absence or presence of morphine; esreboxetine in absence or presence of morphine and/or fluoxetine; fluoxetine in absence or presence of morphine and/or esreboxetine; and duloxetine in absence or presence of morphine and ondansetron - at 75 min post-dosing.

Dose (mg/kg)	[Plasma]_unbound_ (ng/ml)	[Brain]_unbound_ (ng/g)
	Atomoxetine 10 mg/kg	
− Morphine	50.9±5.8	58.2±10.9
+ Morphine 3	57.9±12.5	52.6±14.4
	Esreboxetine 10 mg/kg	
− Fluoxetine - Morphine	33.4±12.2	20.4±14.4
+ Morphine 1	21.0±17.7	12.7±9.9
+ Fluoxetine 1+ Morphine 1	31.2±8.6	25.0±5.8
	Fluoxetine 1 mg/kg	
− Esreboxetine - Morphine	1.73±1.5	1.94±1.2
+ Morphine 1	0.96±0.3	1.36±0.6
+ Esreboxetine 10+ Morphine 1	1.51±0.5	1.55±0.6
	Duloxetine 5 mg/kg	
− Ondansetron - Morphine	4.98±1.4	13.0±1.5
+ Ondansetron 3	3.51±1.2	12.8±4.5
+ Ondansetron 3+ Morphine 1	4.55±0.7	16.5±4.1

n = 3–6 for each group. Data are presented as mean ± standard deviation.

To confirm the hypothesis that inhibition of both NET and SERT is required to enhance the antinociceptive effect of morphine, a fixed-dose combination of esreboxetine (10 mg/kg, IP) and fluoxetine (1 mg/kg, IP) was coadministered with increasing doses of morphine. This fixed dose combination alone reduced flinching behavior by 24±11% in the rat formalin model and was associated with 78±8% NET and 55±3% SERT occupancy, measured *ex vivo* at 75 min post-treatment. The NET and SERT occupancies were similar to those observed with a 10 mg/kg dose atomoxetine (see above). Using a fixed-dose experimental design, the combination of esreboxetine and fluoxetine shifted the morphine dose-response curve to the left, consistent with potentiation of the morphine antinociceptive effect (Fig. 2C; ED_50_ = 0.3 mg/kg with 95% CI: 0.2–0.7). The plasma _unbound_ and brain _unbound_ concentrations of esreboxetine and fluoxetine were not significantly different between rats administered the selective NE or 5-HT reuptake inhibitors alone, and those administered them in combination with 3 mg/kg SC morphine (Table 2). Collectively, these observations suggest a simple drug-drug interaction did not account for the enhanced antinociceptive activity observed following co-administration of morphine and the combination of the NET and SERT inhibitors.

**Figure 3:**
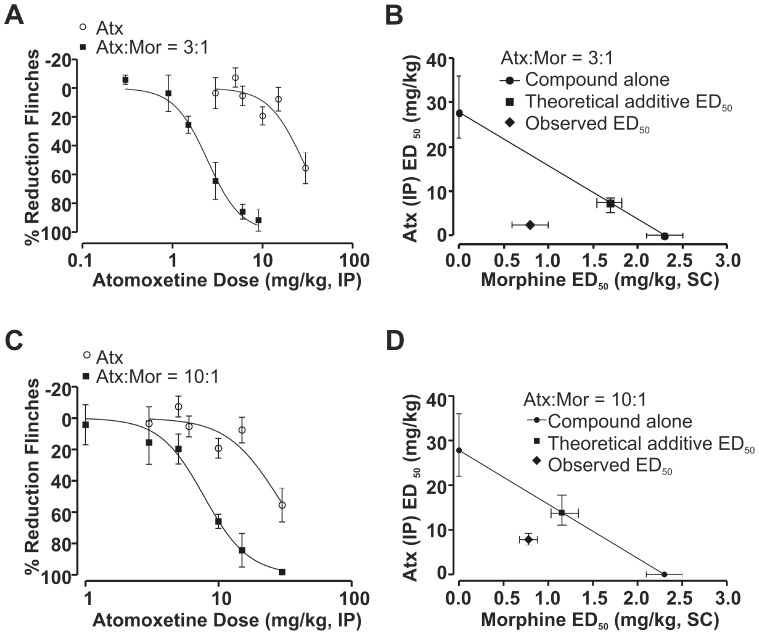
Atomoxetine exhibited antinociceptive synergy with morphine using a fixed-ratio design in the rat formalin model. (A) The dose-response curve of a fixed-ratio of 3 parts atomoxetine (Atx, IP) to 1 part morphine (Mor, SC) leftward shifted relative to the atomoxetine dose-response curve alone (n = 6–12). All data points are shown as mean ± SEM for each group and are expressed as percentage of controls. (B) An isobologram for the combined effects of atomoxetine and morphine in a fixed ratio combination 3∶1. The ED_50_ value for morphine is plotted on the abscissa, and the ED_50_ value for atomoxetine is plotted on the ordinate. The solid line represents the line of additivity and the isobol point (observed ED_50_ value) is located to the left and below the theoretical additive ED_50_ value (with non-overlapping 95% CI). (C) The dose-response curve of a fixed-ratio of concomitant administration of 10 part atomoxetine (IP) to 1 part morphine (SC) leftward shifted relative to the atomoxetine dose-response curve alone (n = 6–16). All data points are shown as mean ± SEM for each group and are expressed as percentage of controls. (D) An isobologram for the combined effects of atomoxetine and morphine in a fixed ratio combination 10∶1. The isobol point (observed ED_50_ value) is located to the left and below the theoretical additive ED_50_ value (without overlapping 95% CI).

Given the enhanced potency observed at fixed-dose ratios of 3∶1 and 10∶1 of atomoxetine to morphine, we next used a fixed-ratio design and isobolographic analysis to confirm antinociceptive synergy. The 3∶1 ratio of atomoxetine to morphine significantly shifted the atomoxetine dose-response curve to the left (Fig. 3A). Isobolographic analysis revealed that the observed ED_50_ value of the 3∶1 combination, 2.4 mg/kg (95% CI: 2.0–3.0), was significantly less than the theoretical additive ED_50_ value of 7.4 mg/kg (95% CI: 6.3–8.2; Fig. 3B). The 10∶1 ratio yielded a similar leftward shift in the atomoxetine dose-response curve (Fig. 3C) and an ED_50_ of 7.8 mg/kg (95% CI: 6.6–9.2) which was significant less than the theoretical additive ED_50_ value of 13.8 mg/kg (95% CI: 11.2–17.8; Fig. 3D).

**Figure 4:**
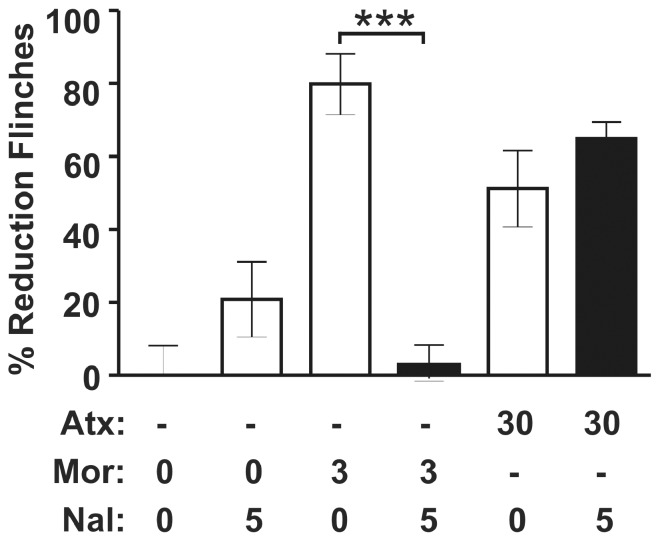
The antinociceptive activity of atomoxetine in the rat formalin model was independent of µ-opioid receptor activation. The µ-opioid receptor antagonist naloxone (Nal, IP, 5 mg/kg), at a dose which effectively blocked morphine (Mor)-induced analgesia in the rat formalin model, did not inhibit atomoxetine (Atx)-induced antinociception (n = 5–7). All values are shown as mean ± SEM for each group and are expressed as percentage of controls. Student’s *t* test, t _(10)_ = 7.668, ***p<0.001.

To evaluate the possibility of a direct effect of atomoxetine on the µ-opioid receptor, naloxone was administered with atomoxetine (10 and 30 mg/kg IP). In the rat formalin model, naloxone failed to attenuate atomoxetine-induced antinociception at a dose (5 mg/kg) that effectively blocked morphine (3 mg/kg)-induced antinociception (t _(10)_ = 7.668, p<0.001; Fig. 4).

**Figure 5:**
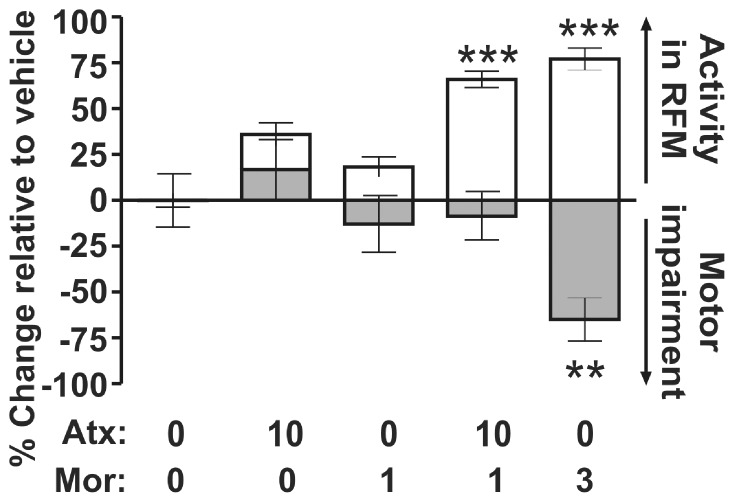
Antinociceptive synergy between atomoxetine and morphine did not reflect impaired motor coordination. The white bars represent the % reduction in the flinching behavior compared to vehicle-treatment in the rat formalin model (n = 10–22), and the grey bars represent the change in latency for rats to fall from an accelerating rotating rod compared to vehicle treatment in the rat RotaRod test (n = 8). All data points are shown as mean ± SEM for each group and are expressed as percentage of controls. Data from one-way ANOVA are as follows: rat formalin model: F _(4, 53)_ = 36.12, p<0.0001; RotaRod: F _(4, 34)_ = 4.604, p = 0.004. Data from the *post hoc* Dunnett’s test follows: **p<0.01, q = 3.265; ***p<0.001, q = 9.258–9.370, compared to vehicle treatment.

To establish that the antinociceptive synergy between morphine and atomoxetine was independent of an effect on motor function, we tested this combination in the RotaRod assay of motor coordination. Co-administration of atomoxetine (10 mg/kg IP) with morphine (1 mg/kg SC) produced a 66±4% reduction in flinching behavior but no impairment of motor coordination (9±13%, p>0.05). By contrast, a 3 mg/kg SC dose of morphine alone, which decreased nociceptive behavior by 77%±6%, was associated with a statistically significant reduction in walking time (65±12%; F (4, 34) = 4.604, p = 0.004, Fig. 5).

### 3.4. Duloxetine Fails to Enhance Morphine-induced Antinociception

**Figure 6:**
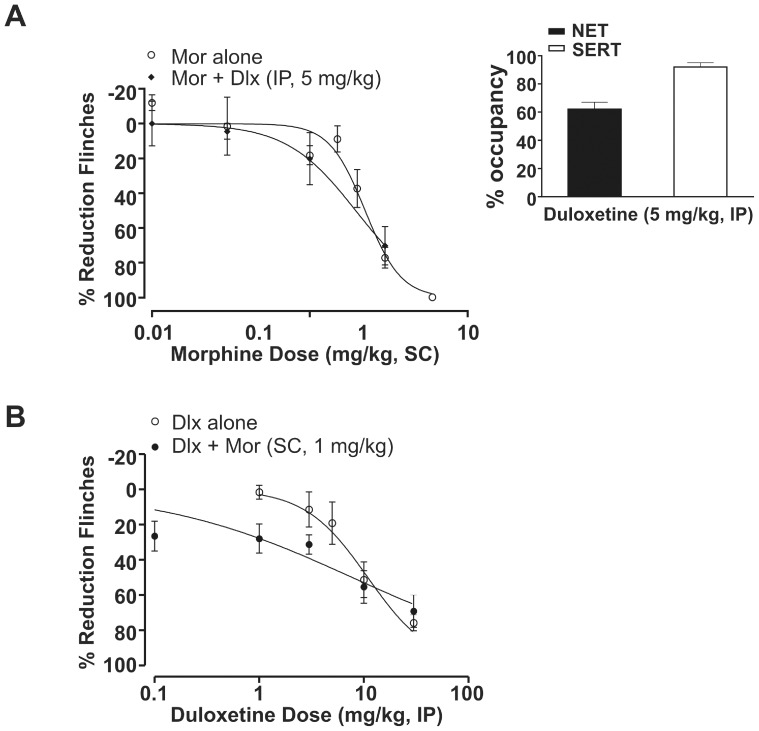
Duloxetine failed to exhibit antinociceptive synergy with morphine in the rat formalin model. (A) Duloxetine (Dlx) at 5 mg/kg failed to shift the morphine (Mor) dose-response curve leftward. Morphine alone: ED_50_ = 2.3 mg/kg (95% CI: 2.0–2.5); morphine+duloxetine (IP, 5 mg/kg): ED_50_ = 2.0 mg/kg (95% CI: 1.3–3.0). All data points are shown as mean ± SEM for each group and are expressed as percentage of controls. Inset (A) Duloxetine (IP) at 5 mg/kg was associated with 62±5% for NET and 92±3% for SERT occupancy measured *ex vivo* at 75 min post-dose. All occupancy data represent mean (± SEM) for each group. (B) A subefficacious dose of morphine 1 mg/kg (SC) failed to left-shift the duloxetine dose-response curve (n = 6–12). Duloxetine alone: ED_50_ = 10.9 mg/kg (95% CI: 8–15); and duloxetine+morphine (SC, 1 mg/kg): ED_50_ = 7.7 mg/kg (95% CI: 4–16).

Duloxetine, a dual monoamine reuptake inhibitor that inhibits both serotonin and norepinephrine transporters, alone produced dose-dependent antinociception in the rat formalin model, with an ED_50_ of 10.9 mg/kg (95% CI: 8–15; Fig. 6B). Duloxetine reduced flinching behavior by 19±12% at dose of 5 mg/kg, IP (Fig. 6B). At this dose, the corresponding transporter occupancies were 62±5% for NET and 92±3% for SERT (Fig. 6A inset), comparable to 3 mg/kg atomoxetine at NET but superior at SERT. Using a fixed-dose experimental design, subefficacious doses of duloxetine, 5 mg/kg IP, failed to shift the morphine dose-response curve leftward (Fig. 6A; ED_50_ = 2.0 mg/kg with 95% CI: 1.3–3.0). Moreover, a subefficacious dose of morphine (1 mg/kg, SC) did not evoke a significant leftward shift in the duloxetine dose-response curve (Fig. 6B; ED_50_ = 7.7 mg/kg with 95% CI: 4–16).

### 3.5. Ondansetron Potentiates the Antinociceptive Response to Duloxetine and Morphine

**Figure 7:**
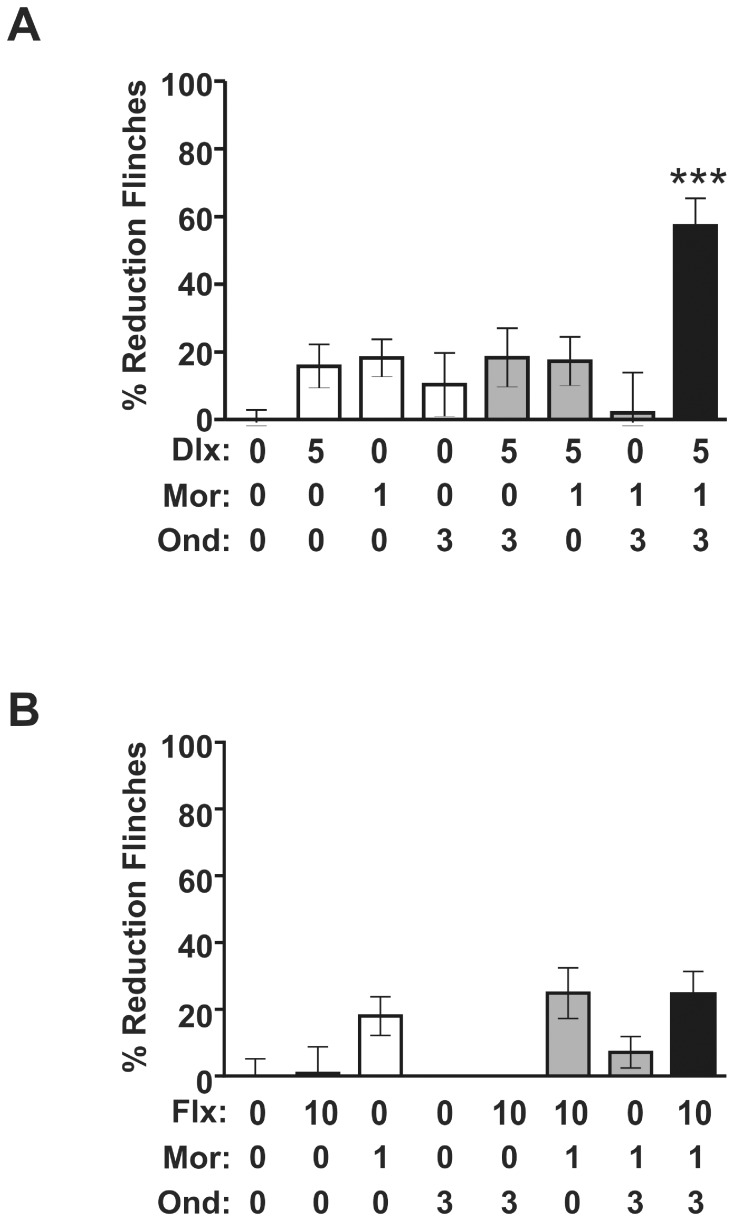
Coadministration of ondansetron potentiates the antinociceptive response to duloxetine and morphine in the rat formalin model. (A) Co-administration of the 5-HT_3_ receptor selective antagonist ondansetron (Ond, IP, 3 mg/kg) potentiates the antinociceptive response to duloxetine (Dlx, IP, 5 mg/kg) and morphine (Mor, SC, 1 mg/kg). Ondansetron alone, or in combination with either morphine or duloxetine, did not exhibit antinociceptive activity (n = 6–19). All data points are shown as mean ± SEM for each group and are expressed as percentage of controls. One-way ANOVA: F _(7, 75)_ = 7.447, p<0.0001. Data from the *post hoc* Newman-Keuls test follows: ***p<0.001, q = 4.956–9.764 for duloxetine+morphine+ondansetron versus the other groups. (B) Co-administration of ondansetron (IP, 3 mg/kg) did not reveal antinociceptive synergy between the SERT selective reuptake inhibitor fluoxetine (Flx, IP, 10 mg/kg) and morphine (SC, 1 mg/kg). Ondansetron alone, or in combination with either morphine or fluoxetine, did not exhibit antinociceptive activity (n = 7). One-way ANOVA followed by *post hoc* Newman-Keuls: p>0.05 morphine versus fluoxetine+morphine; p>0.05 fluoxetine+morphine versus fluoxetine+morphine+ondansetron.

Given the observed synergy between atomoxetine and morphine in the rat formalin model, the lack of synergy with duloxetine (5 mg/kg), which demonstrated comparable NET occupancy to 3 mg/kg atomoxetine, was unexpected. Because there is evidence that 5-HT_3_ receptors participate in 5-HT-mediated descending facilitation of the pain modulatory pathway [34], we co-administered the 5-HT_3_ receptor antagonist, ondansetron (3 mg/kg IP), with a sub-efficacious dose of duloxetine (5 mg/kg IP) and morphine. Ondansetron potentiated the antinociceptive response to duloxetine and morphine (1 mg/kg SC) (F (7, 75) = 7.447, p<0.0001, Fig. 7A). The plasma_ unbound_ and brain_ unbound_ concentrations of duloxetine were not significantly different between rats administered the monoamine reuptake inhibitor alone and those administered the inhibitor in combination with 3 mg/kg IP ondansetron and 1 mg/kg SC morphine (Table 2). Furthermore, coadministration of ondansetron did not change duloxetine SERT and NET occupancy measured *ex vivo* (the corresponding transporter occupancies were 62±5% for NET and 92±3% for SERT for duloxetine alone, and 55±6% for NET and 89±4% for SERT for duloxetine with ondansetron). These observations suggest that a simple drug-drug interaction is unlikely to account for the enhanced antinociceptive activity observed. Ondansetron alone, or in combination with either morphine or duloxetine alone, did not exhibit antinociceptive activity (Fig. 7A). By contrast, co-administration of ondansetron (3 mg/kg IP) failed to potentiate the antinociceptive response to the SERT selective reuptake inhibitor fluoxetine (10 mg/kg, IP) and morphine (1 mg/kg SC) (Fig. 7B). Coadministration of ondansetron did not change fluoxetine SERT and NET occupancy measured *ex vivo* (the corresponding transporter occupancies were 6±12% for NET and 89±5% for SERT for fluoxetine alone, and 7±5% for NET and 87±3% for SERT for fluoxetine with ondansetron). Collectively these data support the hypothesis that concurrent NET and SERT inhibition is necessary for a monoamine reuptake inhibitor to exhibit antinociceptive synergy with morphine.

## Discussion

The present study tested monoamine reuptake inhibitors with distinct pharmacological profiles, in conjunction with measurements of antinociception in the rat formalin model and *ex vivo* transporter occupancy, to explore the relative contributions of NET and SERT inhibition to antinociceptive synergy with morphine. We showed that selective inhibition of NET (e.g., esreboxetine), or SERT (e.g., fluoxetine), alone is insufficient for antinociceptive synergy with morphine. However, atomoxetine, a monoamine reuptake inhibitor that engages more NET than SERT, exhibited synergy with morphine at doses that yielded significant NET occupancy (≥67%) and modest SERT occupancy (∼35 to 64%). Similarly, a fixed-dose combination of esreboxetine and fluoxetine which achieves comparable levels of CNS transporter occupancy potentiated the antinociceptive response to morphine. In contrast, duloxetine, a monoamine reuptake inhibitor that engages more SERT than NET, did not exhibit antinociceptive synergy with morphine at a dose which produced comparable NET (62%) and near-maximal (>85%) occupancy of SERT. Thus, monoamine reuptake inhibitor and morphine-mediated antinociceptive synergy requires dual engagement of both NE and 5-HT transporters, but excessive SERT occupancy may mask the synergistic interactions between these antinociceptive systems.

To our knowledge, these results represent the first quantitative demonstration that the balance between NET and SERT inhibition for a parenterally administrated monoamine reuptake inhibitor can influence the synergistic interaction with morphine in the rat formalin model.

Our findings are consistent with numerous other studies that have reported synergy and/or additivity between morphine and agents that modulate 5-HT or NE pathways [13], [14], [16]–[19]. Preclinically, in addition to the rat formalin model, synergistic interactions between monoamine reuptake inhibitors and morphine have also been observed in mouse tail-flick, rat thermal paw withdrawal and in preclinical pain models of postoperative pain [17]–[19]. Recently, Schroder *et. al.* [35] demonstrated a synergistic interaction between NET inhibition and µ-receptor agonism in the low-intensity tail-flick and spinal nerve ligation rat models based on isobolographic analysis of estimated receptor/transporter occupancy for tapentadol. The predicted antinociceptive synergy with the dual mechanism tapentadol contrasts with our -conclusion that modest SERT engagement is required for antinociceptive synergy with morphine. The conflicting results could reflect different occupancy requirements in the rat formalin model compared with other models. It is also possible that the isobolographic analysis based on occupancy estimates calculated from tapentadol’s brain concentration could yield a different interpretation than one based on direct *ex vivo* occupancy measurements from rats monitored for nociceptive behavior, as in our current study. In clinical settings, desipramine, a TCA that inhibits NET preferentially over SERT, enhances morphine analgesia in post-operative dental pain patients [13]. However, the clinical reports that duloxetine can reduce morphine consumption in both acute [14] and chronic [16] pain populations appear to be at odds with our preclinical finding of a lack of synergy between duloxetine and morphine and are worthy of further discussion. Possible discrepancies between the clinical and preclinical observations with duloxetine include dose – occupancy estimates and the technical limitations of each setting. As reported by positron emission tomography, at the therapeutic dose of 60 mg duloxetine achieves near-maximal occupancy of SERT, comparable to that observed in our current study [36]. While the absolute level of NET occupancy achieved at therapeutic doses of duloxetine has not been reported, duloxetine likely engages NET at the clinical exposures [31], [37]. The difference between the clinical and the current preclinical findings with duloxetine may reflect different transporter occupancy requirements and/or differences in intrinsic NE and/or 5-HT tone in the preclinical model versus clinical pain states. An even simpler explanation of the potential discrepancy is that it is difficult to discriminate between additive and synergistic interactions in the clinical setting. As such, it is conceivable that the duloxetine clinical data represent simple additive effects of two analgesics with distinct mechanisms of action. Similarly, in the rat formalin model higher doses of duloxetine, which were antinociceptive in the absence of morphine, exhibited additivity with morphine (data not shown).

The neural substrates and corresponding mechanisms underlying a synergistic interaction between monoamines and opioids remain largely unknown. It has been shown that when a noxious stimulus is presented alone (e.g., formalin injection in the paw), activation of the endogenous pain modulatory pathway leads to a bilateral increase in the concentrations of both NE and 5-HT in the dorsal horn of the spinal cord [1], [24]. Coadministration of systemic morphine, in the presence of noxious stimulation, further increases the neuronal activity of brainstem noradrenergic, but not serotonergic, neurons [38]. Electrophysiological [22] or regional shRNA interference combined with behavioral [23] techniques have demonstrated that activation of descending 5-HT containing neurons from the rostral ventromedial medulla (RVM) is neither necessary, nor sufficient, for µ-opioid receptor agonist-induced analgesia. Furthermore, interactions between α_2_-adrenoceptors and µ-opioid receptors at the spinal level have also been reported [39], [40]. In the clinical arena, intrathecal clonidine is widely used in combination with morphine to afford equivalent pain-relief post-operatively, but with reduced morphine consumption (i.e., morphine-sparing) [41], [42]. The use of reuptake inhibitors with an appropriate balance of NET and SERT may provide an opportunity for a systemically-administered drug with opioid-sparing properties analogous to those of the intrathecally administered α_2_-adrenoceptor agonists.

While the contribution of descending noradrenergic and serotonergic inhibitory pain pathways may be important for the antinociceptive synergy between monoamine reuptake inhibitors and morphine, the contribution of descending pathways that facilitate pain transmission cannot be ignored. Indeed, a growing body of literature suggests that sustained activation of these circuits may underlie some states of chronic pain [43]–[45]. Thus, serotonergic signals relayed from the RVM in the brainstem can produce anti- or pro-nociceptive effects depending on the particular 5-HT receptor subtype recruited [34], [46]. It has been shown that activation of 5-HT_3_ receptors increases the open probability of voltage-gated calcium channels, which in turn enhances excitatory neurotransmitter release to exert a pro-nociceptive effect [47]. However, Paul and colleagues reported that spinal injection of 5-HT_3_ receptor antisense attenuated intrathecally-administered 5-HT-mediated antinociception in the mouse tail flick model, which would suggest an antinociceptive effect from activation of spinal 5-HT_3_ receptors [48]. The apparent discrepancy between these results and the present observations may reflect a differential contribution of spinal and supraspinal 5-HT_3_ receptors in the respective pain models. For example, a large body of literature from the rat formalin model supports the hypothesis that activation of both spinal and supraspinal 5-HT_3_ receptors is pronociceptive [49]–[51]. In the present study, coadministration of the 5-HT_3_ receptor selective antagonist, ondansetron, potentiated the antinociceptive response to duloxetine and morphine at doses that failed to show activity alone. One interpretation of the present data is that activation of 5-HT_3_ receptors in the descending serotonergic facilitatory pathway prohibits synergistic antinociceptive interactions between monoamine reuptake inhibitors and morphine in the rat formalin model. However, as discussed above, the observation that the NET-selective reuptake inhibitor, esreboxetine, did not enhance the antinociceptive response to morphine suggests that a critical level of SERT inhibition, and consequent elevation of spinal and/or supraspinal 5-HT levels, is necessary. We hypothesize that a modest level of SERT inhibition may be required to enhance descending inhibitory serotonergic pathways, while near-maximal SERT inhibition enhances descending facilitatory serotonergic pathways which, through activation of 5-HT_3_ receptors, preclude a synergistic interaction between a monoamine reuptake inhibitor and morphine in the rat formalin model.

When testing a pair of drugs for potential synergy, additivity or antagonism, both “fixed-dose” and “fixed-ratio” experimental designs are commonly used [33], [52]–[56]. A “fixed-dose” design tests the dose-response curve for drug A in the presence and absence of a fixed-dose of drug B (usually subefficacious on its own), whereas a “fixed-ratio” design tests a series of dilutions of a constant ratio of drug A to B [57], [58]. The present study used both regimens to explore, and subsequently confirm, potential synergistic interactions between atomoxetine and morphine. Previous studies have highlighted how dose ratios can influence the apparent synergistic, additive or even antagonistic interactions between two drugs [59]–[61]. This phenomenon may explain the shallow dose-response curve observed in the fixed-dose combination studies (Figs. 2 & 6) and the observation that some ratios of the combination were synergistic and others only additive. In the present study, the fixed-dose design was used to guide optimal ratio selection for atomoxetine and morphine and provide insight into the SERT and NET occupancy associated with morphine potentiating effects, whereas the fixed-ratio design was used to confirm antinociceptive synergy between atomoxetine and morphine. Ultimately, the choice of a fixed-dose versus a fixed-ratio design may depend more on the intent of the study. For instance, a preclinical study using a fixed-dose design may be more relevant for clinical settings in which analgesics with different mechanisms are combined with intravenous morphine peri-operatively to reduce the morphine burden [14], [41], [42]. On the other hand, fixed-ratio regimens would be useful for designing or optimizing novel dual-mechanism drugs that possess both opioid and noradrenergic properties, as exemplified by tapentadol. While both approaches embrace the concept of targeting multiple pain pathways simultaneously to achieve superior antinociception without exacerbating side effects, each approach has limitations. For example, the coadministration of multiple analgesics carries the potential risk of an increased xenobiotic load and requires that the kinetic profiles of the individual analgesics be appropriately matched. Conversely, a novel analgesic that exhibits the desired dual pharmacology presents additional design challenges and offers limited flexibility to alter the relative levels of modulation of the respective targets.

Several features of the present study may limit the interpretation of our findings. For example, a likely site of action for monoamine reuptake inhibitors is the spinal cord, yet we measured transporter occupancy only in the rat cortical homogenates. Under circumstances in which we measured NET and SERT occupancies in both brain and spinal cord, the data for the two regions were comparable (Theravance, Inc., unpublished data). Nevertheless, the relationship between transporter occupancy and neurotransmitter levels measured at multiple putative sites of antinociceptive action remains worthy of investigation. Moreover, in considering the potential for synergistic interactions with respect to side effects that accompany administration of monoamine reuptake inhibitors or morphine, we assessed only motor impairment. As we did not assess other potential µ-opioid receptor agonist-induced adverse effects (e.g. respiratory depression, bowel dysfunction, etc.), we cannot speculate on the extent to which NET and SERT inhibition would influence the overall tolerability profile of the drug combinations studied.

In summary, the present study has demonstrated that NET inhibition is necessary, but not sufficient, for antinociceptive synergy between a monoamine reuptake inhibitor and morphine in the rat formalin model. Modest SERT engagement is required to observe synergy between NET inhibitors and opioids. At near-maximal levels of SERT inhibition, as observed with duloxetine, activation of 5-HT_3_ receptors in descending facilitatory serotonergic pathways likely precludes manifestation of a synergistic interaction with morphine. Translation of these preclinical observations to clinical pain states awaits rigorous clinical evaluation of the morphine-sparing properties of dual reuptake inhibitors with distinct NET/SERT selectivity profiles.
